# Usefulness of disease surveillance data in enhanced early warning of the cholera outbreak in Southwest Cameroon, 2018

**DOI:** 10.1186/s13031-023-00504-1

**Published:** 2023-02-07

**Authors:** Reine Suzanne Mengue Kadia, Benjamin Momo Kadia, Christian Akem Dimala, Andrew E. Collins

**Affiliations:** 1 Department of Geography and Environmental Sciences, Faculty of Engineering and Environment, Northumbria University, Newcastle, UK; 2 Health Education and Research Organization (HERO), Buea, Cameroon; 3 Department of Clinical Sciences, Liverpool School of Tropical Medicine, Liverpool, UK; 4 Health and Human Development (2HD) Research Network, Douala, Cameroon; 5 Department of Medicine, Reading Hospital, Tower Health System, West Reading, PA USA

**Keywords:** Cholera, Conflict, Disease surveillance, Early warning, Routine data, Cameroon

## Abstract

**Introduction:**

This study assessed the timeliness and completeness of disease surveillance data for early warning of the cholera outbreak during the socio-political crisis of Southwest Cameroon in 2018. It determined how routine integrated disease surveillance and response (IDSR) data was used for preventative actions and the challenges faced by key health staff in IDSR based decision-making.

**Methods:**

This was a mixed-methods study conducted from June 1st to September 30th 2021. District Health Information System 2 (DHIS2) data from January 2018 to December 2020 for the Southwest region of Cameroon were analysed using simple linear regression on EPI Info 7.2 to determine a potential association of the sociopolitical crisis with timeliness and completeness of data. Qualitative data generated through in-depth interviews of key informants were coded and analyzed using NVivo 12.

**Results:**

During high conflict intensity (2018 and 2019), average data timeliness and completeness were 16.3% and 67.2%, respectively, increasing to 40.7% and 80.2%, respectively, in 2020 when the conflict intensity had reduced. There was a statistically significant weak correlation between reduced conflict intensity and increased data timeliness (R^2^ = 0.17, *p* = 0.016) and there was also a weak correlation between reduced conflict intensity and data completeness but this was not statistically significant (R^2^ = 0.01, *p* = 0.642). During high conflict intensity, the Kumba and Buea health districts had the highest data timeliness (17.2% and 96.2%, respectively) and data completeness (78.8% and 40.4%, respectively) possibly because of proximity to reporting sites and effective performance based financing. Components of IDSR that should be maintained included the electronic report aspect of the DHIS2 and the supportive supervision conducted during the outbreak. Staff demotivation, the parallel multiplicity of data entry tools, poor communication, shortage of staff and the non-usability of data generated by the DHIS2 were systemic challenges to the early alert dimension of the IDSR system. Non–systemic challenges included high levels of insecurity, far to reach outbreak sites and health personnel being targeted during the conflict.

**Conclusion:**

In general, routine IDSR data was not a reliable way of providing early warning of the 2018 cholera outbreak because of incomplete and late reports. Nonetheless, reduced conflict intensity correlated with increased timeliness and completeness of data reporting. The IDSR was substantially challenged during the crisis, and erroneous data generated by the DHIS 2 significantly undermined the efforts and resources invested to control the outbreak. The Ministry of Public Health should reinforce efforts to build a reporting system that produces people-centered actionable data that engages health risk management during socio-political crises.

## Introduction

Cholera is an acute diarrhoeal bacterial disease caused by a toxin produced by *Vibrio Cholerae* [1]**.** For this pathogen to be transmitted rapidly in the human population, environmental conditions with seasonal changes have to coincide with poor sanitation and hygiene, increased population displacement, breakdown in infection control measures, and overcrowded settings [1]. Conflicts causing complex emergencies combine these conditions leading to a high risk of cholera epidemics [1–3]. Cameroon has experienced several cholera outbreaks since 1971 [4], with the most recent epidemics originating from the Southwest region (SWR). Between 2011 and 2015, there were 1041 registered cholera-related deaths [5].

In October 2016, a socio-political crisis started in the Northwest and Southwest regions of Cameroon and the conflict continues to date [6, 7]. The crisis has caused over 500,000 people to be displaced, and an estimated 1800 people have been killed [7]. The fleeing of health care professionals from rural areas and their site of work, destruction of infrastructure, and lack of resources destabilized the health care system in the Southwest region of Cameroon [8]. The destruction of infrastructure including health, food insecurity, lack of potable water, and widespread insecurity, led to the 2018 cholera epidemic in the SWR of Cameroon [1].

Early warning systems provide a framework for timely and effective response to a disaster and in the public health sector, World Health Organisation (WHO) and the International Health Regulations have adopted the Integrated Disease Surveillance and Response (IDSR) System. The IDSR provides a standard guideline for monitoring, identifying, notifying, and RESPONDING to disease outbreaks. The final results are meant to inform decisions [9]. However, the IDSR system that monitors infectious diseases is not built to be implemented in conflict zones, even though most underdeveloped countries use this system in both non-conflict and conflict environments. Therefore, it is essential to flag functional areas of the IDSR system and non-useful aspects of the IDSR system in a conflict setting, in order to render the IDSR system more efficient. In Cameroon, cholera is one of the priority diseases monitored by the IDSR and researchers have explored the usefulness of the IDSR system in resource-constrained settings but there is paucity of information on its usefulness in resource constrained settings with protracted conflicts as in the SWR of Cameroon. This study sought to assess the average data completeness and timeliness rates for health districts that reported cholera cases between 2018 and 2020, assess the association of the sociopolitical conflict with the early warning of the cholera outbreak (timeliness and completeness), determine how the routine IDSR data was used for effective early warning of the cholera outbreak in the SWR during the sociopolitical conflict and determine the challenges faced by key health staff in utilizing IDSR data at the regional and district levels for decision-making during the 2018 cholera outbreak.

## Material and methods

### Study design

This study adopted a mixed-methods approach: a retrospective analysis of secondary (routine) data from the DHIS2 and analysis of primary data from in-depth key informant interviews. A combination of the Technical Acceptance Model (TAM) and the DeLone and Mclean (D&M) models was used to determine if the routine IDSR data generated by the DHIS 2 provides the information used to make decisions that will ultimately reduce epidemic alert time [10, 11]. User experience of the system throughout the data processing cycle was explored to determine if the data generated by the DHIS 2 could inform decisions for the early alert of the outbreak.

### Study period and setting

**Fig. 1:**
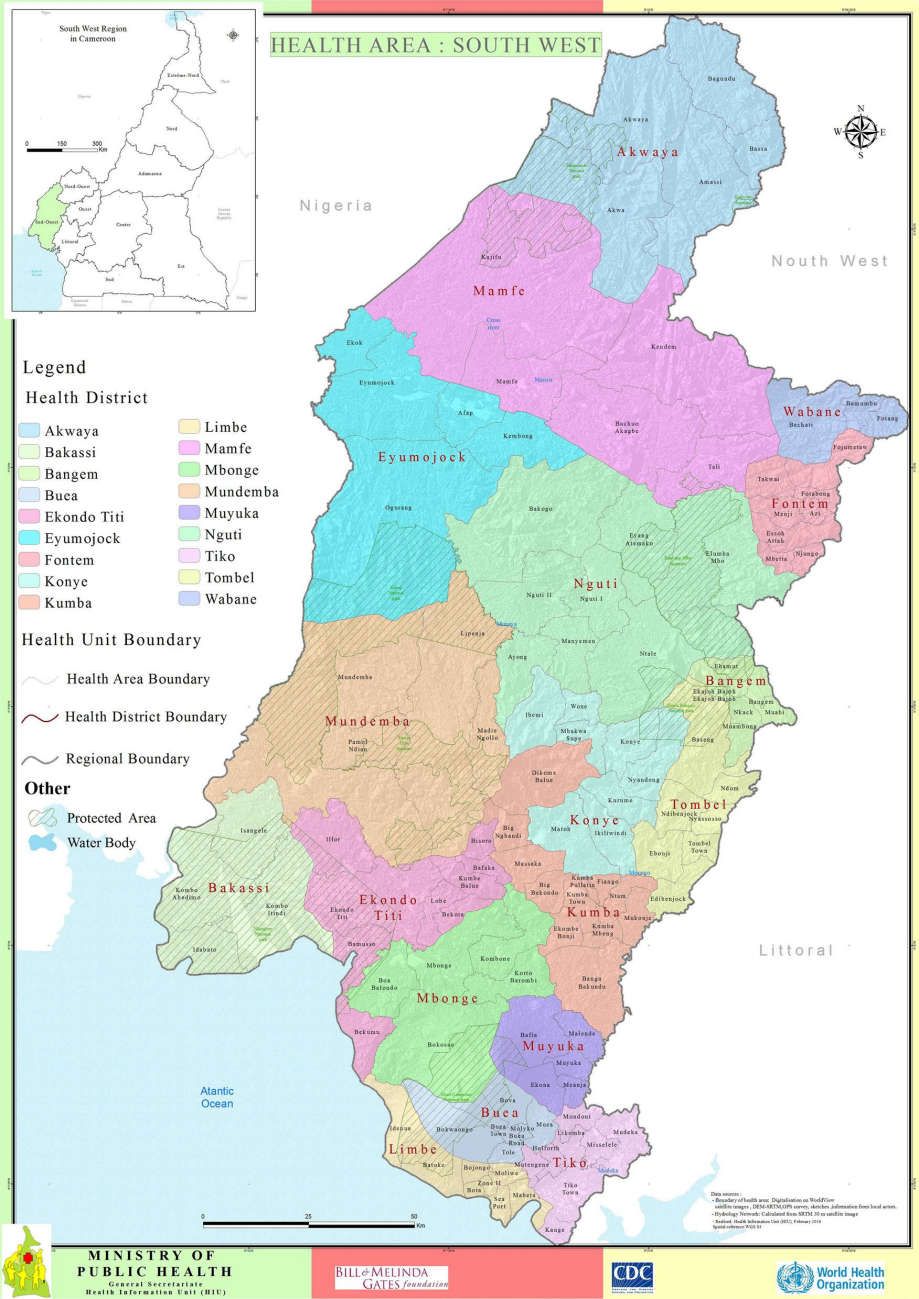
Map of Southwest Region showing different health districts

The Southwest region of Cameroon has an estimated population of 1,899,941. In 2016, a civil crisis broke out in the region and was considered by local authorities to have contributed to the cholera outbreak. The Southwest region has 18 health districts and 126 health areas, the largest of which is Kumba Health district. Figure 1 shows the delimitation of each district, its health areas, and health units. The retrospective phase of the study was done from June 1st to September 30th 2021 (4 months), focusing on the usefulness of the IDSR routine data in the early warning of the 2018 cholera epidemic. Secondary data was collected from the DHIS2 for the period of January 2018 to December 2021 for all health facilities that reported at least one suspected case of cholera in the Southwest Region.

Reporting in the IDSR system is structured such that data moves from the community to the national level. Community health workers are responsible for case identification at the community level, while the disease surveillance focal person of each health facility conducts case identification and reporting to the district health service (weekly and monthly). These focal persons tally the cases using medical records in their respective health facilities. In the most recent National IDSR guideline, 18 diseases and events required immediate reporting, including cholera (Table 1). For cholera, a district IDSR focal person collects, synthesizes, and reports district data assembled from health facilities. From the district level, reporting is done by the district disease surveillance focal person enters the IDSR data into the DHIS 2 which Cameroon adopted in 2014 [12]. The centralized IDSR data is stored in a server managed by the national health information centre (NHIC). However, cholera outbreaks are managed by the Cameroon Ministry of Health [13].

**Table 1:** Priority diseases of epidemic prone potential for immediate reporting

1—Anthrax	10—Rabies (confirmed cases)
2—Chikungunya	11—Measles
3—Cholera	12—Serious Acute Respiratory Syndrome (SARS)
4—Dengue	13—Acute hemorrhagic fever (Ebola, Marburg, Lassa Fever, RVF, Crimean-Congo)
5—Dracunculiasis	14—Neonatal tetanus
6—Yellow fever	15—Smallpox
7—Influenza due to new subtype	16—Any public health event of international concern (infection, zoonosis, food borne infection, chemical, radio nuclear contamination or due to an unknown condition)
8—Acute Flaccid Paralysis (AFP)	17—Meningitis
9—Plague	18—Bloody diarrhoea

### Data sources and measurement

As required by the national guideline, a case of cholera is defined as any person presenting with watery diarrhea with or without vomiting or signs of dehydration. Outside an outbreak, the definition refers to the case of a person > 5 years experiencing severe dehydration or death following acute diarrhea. Finally, a confirmed case is a laboratory stool identification of Vibrio Cholerae [13]. If there is at least one confirmed case of cholera, a cholera outbreak is declared.

**Timeliness** of cholera weekly reports refers to the total number of reports submitted on time divided by the total expected reports expressed as a percentage. **Completeness** of cholera weekly reports refers to the total number of reports submitted regardless of submission time, divided by the total number of reports expected expressed as a percentage. Given that the unit of work for this study is in years, the average timeliness and completeness were calculated and expressed in percentages. **Conflict** refers to armed conflict between the state and another party with at least 250 deaths within a given period [14, 15]. Conflict intensity for each year from 2018 to 2020 was classified as high, medium or low based on the updated World Bank’s classification [15]. In this study, high intensity refers to deaths greater than 250 in a year, and medium intensity to deaths between 25 to 149 per year.

Weekly IDSR data was extracted from the DHIS 2 in June 2021 from the Ministry of Public Health National online Server. As reported by the DHIS, data completeness was the number of reports received against the number of reports expected from a given health facility in the health district. The timeliness of reporting was calculated by using the number of health facilities that submitted their reports on time to the DHS. For good timeliness and completeness, the rates should approach 100%.

Qualitative data was collected from two key informants. One of them was from the Kumba Health District (KHD), the largest district in the region, that had reported suspected cholera cases at the time of the 2018 cholera outbreak. The other informant was from the Southwest Regional Delegation for Public Health (RDPH), where all regional data collected during the outbreak period was submitted. Additionally, the KHD was one of the functioning health districts during the conflict in the region. Therefore, this study assumes that understanding the usefulness of the routine IDSR data in the management of the cholera outbreak during the conflict can best be observed from how this health district managed it at their level. Moreover, the challenges of using the IDSR routine data faced by this district can be looked into as a case of an outbreak in a conflict zone. Key informants at the KHD level included the district disease surveillance focal person at the time of the outbreak, and the health facility disease surveillance focal person of a health facility that was functional during the crisis, as assessed by International Medical Corps in 2018 [6]**.** Meanwhile, the regional disease surveillance focal person was interviewed at the RDPH. This approach was expected to provide insights into avoidable challenges and improve the timeliness and completeness of data during an outbreak in a conflict setting. Due to the Covid-19 pandemic, all interviews were conducted through phone after administering a consent form to the interviewees and digitally recorded for transcription—these interviews aimed at understanding IDSR implementation challenges in a conflict zone during a cholera outbreak.

### Ethics approval and consent to participate

This study was approved by the Northumbria University Ethics Committee and the Cameroon National Ethics Committee.

### Data management and analysis

Quantitative data regarding all reported cases of Cholera was extracted from DHIS 2 and exported into Microsoft Excel. An entry sheet was created in EPI Info 7 to adapt the data to obtain correlation and association relationships between variables. The data were stratified into different years to determine the pattern evolution over time compared to the ongoing sociopolitical crisis. A simple linear regression was used to determine the correlation between timeliness, completeness, and the ongoing sociopolitical crisis. The threshold for statistical significance was set at *p* = 0.05. General timeliness and completeness were compared to the standard 80% recommended by the national guideline. Qualitative data were imported into NVivo 12 and a thematic approach was used to analyze the data. The data were categorized into three thematics. Codes were developed to aid deductive analysis. The performance of each of the aforementioned core functions was compared to the national guideline requirements to see if the IDSR system was optimum during a public health emergency in a conflict. Codes were generated to identify challenges in providing valuable data to reduce the cholera outbreak alert time. All the data collected and analyzed were stored in a password-protected laptop with the password detained only by the investigators.

## Results

### Descriptive analysis

Of the 18 health districts, eight health districts were eligible for this study because they reported at least one case of cholera during the period under investigation. Thirty-one health facilities reported the data extracted from DHIS2. Table 2 shows the general case fatality rate captured by DHIS2 during the study period. With a total population of 1,222,917 for the eligible health districts, 417 suspected cholera cases were reported against 23 deaths, giving a case fatality rate of 5.5%. The Limbe health district had the highest case fatality rate (20%), although the number of cholera cases reported from the health district was inferior to those of the Bakassi, which reported more suspected cases.

**Table 2:** General information on affected populations

N0	Health District	Number of Health Facility	Catchment Population	Number of Suspected cholera cases	Number of Cholera deaths	Case fatality rate (%)
1	Bakassi	09	35,911	257	15	5.8
2	Buea	37	180,843	5	1	20
3	Ekondo Titi	16	58,616	8	0	0
4	Kumba	35	379,114	1	0	0
5	Limbe	35	206,887	67	6	8.9
6	Mamfe	24	85,486	0	0	0
7	Muyuka	23	118,276	1	0	0
8	Tiko	23	157,784	74	1	1.5
	Total	202	1,222,917	417	23	5.5

### Average data completeness and timeliness rates for health districts that reported cholera cases

**Fig. 2:**
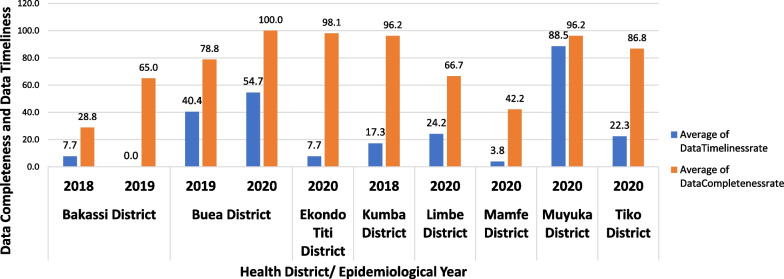
Average data timeliness and completeness as reported during the 2018–2020 cholera outbreak

The transmission of routine IDSR data from the peripheral level to the central level where decisions are made was prolonged and data timeliness and completeness tended to be poor (Fig. 2). During the 2018–2020 period, the average data completeness as reported by eight health districts and 18 health facilities was at 71.9%, while the timeliness was at 20.3%. Bakassi Health District had the lowest completeness (28.8% in 2018) but the highest case notification rate (257 suspected cases, 8 deaths). An average of 79.9% of reports across the districts were not received on time. The highest frequency of timeliness of the reports was in Muyuka Health District (88.5% in 2020), while the lowest frequency of timeliness was in the Bakassi health district (0% in 2019).

### Association of the socio-political crisis with early warning of the cholera outbreak

**Fig. 3:**
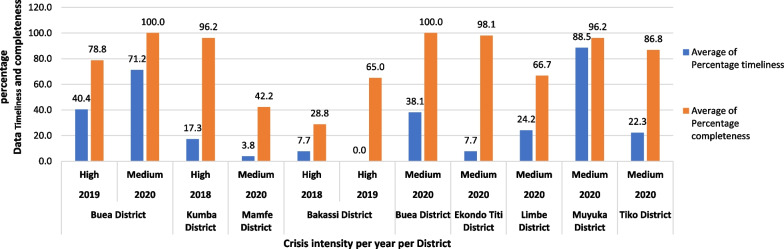
Trends in average data completeness and timeliness against crisis intensity

Three health districts reported 259 suspected cholera cases and 15 cholera-related deaths during high conflict intensity (2018 and 2019). An average of 16.3% and 67.2% of reports were timely and complete, respectively, across these districts. Meanwhile, seven health districts reported 158 suspected cholera cases and eight cholera-related deaths during the medium intensity conflict period (2020). The corresponding average data timeliness and completeness rates for the medium conflict intensity period were 40.7% and 80.2%, respectively. There was a statistically significant weak positive correlation between reduced conflict intensity and increased data timeliness (R^2^ = 0.17, *p* = 0.016) and there was a weak positive correlation between reduced conflict intensity and increased data completeness but this was not statistically significant (R^2^ = 0.01, *p* = 0.642). Figure 3 shows how the conflict intensity affected reporting. Reporting was better during medium conflict intensity than during high conflict intensity. Some outliers, such as the Kumba and Buea health districts, reported comparatively high timeliness and completeness rates despite the high-intensity conflict.

### Use of routine IDSR data for effective early warning of the cholera outbreak

#### Case definition

One key informant said that at the time of the outbreak, one measure was to adapt the case definition at the community level and the health facility level so that it could be more sensitive and capture as many suspected cases as possible.

**Table:** 

“*There were case definitions at all levels. At the community level, when you see someone with profuse diarrhoea you should notify at his or her catchment health facility. There were case definitions at the level of the health facility too.” CBH MoH*	

#### Practice case definition at the time of the outbreak

Both the community and the health facility level staff had a good knowledge of the various case definitions, but there was a problem of implementation due to the ongoing crisis, which caused high insecurity and demotivated health staff as they were targeted during the conflict. The other key informant mentioned that it was difficult to follow the national guidelines such as the case definitions during the outbreak because of high levels of insecurity. The informant said that usually, compared to the 2010 cholera outbreak where there was huge field support from the central level, the 2018 cholera outbreak was characterized by fear, insecurity, and the inability of community health workers to do active community searching of cases.

**Table:** 

“*Since we are government workers, and we are targeted so the army is supposed to go with us because sometimes there are shootings, and this demotivates people to go and work.”* *In the Southwest I have experienced two cholera outbreaks. In 2010 and Yaoundé came and helped me and the rules of IDSR were respected especially the case definition but in 2018 it was not possible to observe the guidelines because of the insecurity” EPDRFP MoH*	

### Disease surveillance tools

During an outbreak, the reporting tools are essential because the ease with which they can be handled provides an opportunity to gain time and enhance early warning of epidemics. Both key informants said three tools were used during. The DHIS 2 software was used at the health facility, district, regional and central levels, and the excel database used paper-based reports to key in data. The paper-based reports were used at the health facility level and transmitted to the health district level. This was found at the district and regional levels and was used concurrently with the DHIS tool.

**Table:** 

*“In 2010 we had the Excel data, and health facilities filled the paper based weekly and monthly disease surveillance. However, in 2018 in addition to the Excel database the DHIS 2 was introduced. The DHIS has a problem that not all health facilities are filling the tool so. The rule is that Health facilities fill directly into the DHIS on Monday before 12:00 noon and the entire world can then access the data but in 2018 we could wait for more than 24 h to see the data”. EPDRF MoH*	

### Reporting

#### User-friendly reporting system

Three tools were used for reporting, of which one had newly been introduced. Each event had to be reported three times through the system. Also, the health district conducted regular supervisions during the outbreak and ceased the opportunity to collect paper-based reports rather than wait for the facilities to report on Monday by noon.

### Challenges faced by key health staff in utilizing IDSR data at the regional and district levels for decision making during the 2018 cholera outbreak

**Table:** 

*“Especially in DHIS 2, in 2018 we had two databases. The Excel database and the DHIS database because it was new in the system. The government wanted both systems to be computerized data. That is how we were working in the context and in the two Anglophone crises. Not all health facilities can fill the information on Monday before 12. In 2018, we could wait for over 24 h to have the information. Supervisions were carried out in the health facility by the health district and partners, and it was an opportunity to collect the data. Therefore, the district does not sit and wait for health facilities to come give them data; they actually go down to the health facility to collect some of the data. So, you make it very easy also for the regional level and the centre level to have the data.” CBH MoH*	

#### Report and data quality

The key informants both said that three reporting systems were used for reporting the same information. One was new, and users did not find it easy to use (DHIS 2). In addition, the informants said that the DHIS 2 had data quality issues, that the data it produced could not be utilized because it was erroneous. The data entered was corrected at the district, regional and central levels, but the quality was poor because not all the data was cleaned.

### Challenges faced by key health staff in utilizing routine IDSR data at the regional and district levels for early warning during the 2018 cholera epidemic and what do they suggest for improvement.

**Table:** 

*“If you access the DHIS 2 platform and see the epidemiological situation of the country you would not come to Cameroon because the data shows that we have plague, Ebola and all of that from the DHIS because of so much erroneous data and people do not take their time to enter the data because they are not motivated.” EPDRF MoH*	

#### Implementation

##### Using IDSR data for decision making

The Epidemic Prone Disease Regional Focal person (EPDRF) said that the erroneous nature of the data in the DHIS 2 did not allow it to be used for decision-making. Moreover, because the reporting tools contained the same information, usually, it was not used for decision-making because it did not give an accurate picture of what was going on.

**Table:** 

*“Even in 2021, we cannot use the DHIS 2 data for decision making. However, since 2019 we gather data managers, work with them and train them on DHIS 2 to ameliorate the performance and the situation. We cannot use it for decision making because it is erroneous, because in a district out of 30 health facilities only two facilities can report while some will not report or the report will not be on time.”* EPDRF MoH

- *Useful aspects of IDSR in the early warning of the cholera outbreak*

At the health district level, the key informant mentioned that regular supervision with the MoPH partners was to ensure that quality work was done and reduced the data transmission time.

At the regional level, the supervision, especially the one done in Bakassi during the onset of the outbreak, permitted the staff to experience the challenges to early submission of data and target interventions to resolve the problem.

**Table:** 

*“We cannot really talk about advantage, but what I can say is that we in Buea have light and internet and all that, and me going there was an experience. To know that the person who sends the report late or does not send at all, I realized that if I had to take a decision at my level … the difficulties that they are facing and when communicating with the central level. I tell them that these are the difficulties that they face because we have means that others don’t have. However, even in Buea we have difficulties.”* EPDRF MoH

b. *Non-useful aspects in the early warning of the outbreak*

Both key informants highlighted the difficulty of using paper-based reports; the transmission time was more prolonged than with the electronic version. Also, all the three reporting tools primarily depended on the paper-based report.

**Table:** 

*“The documentation at the level of the health facility was not really good. So this information that was not well filled can be sent to the district, regional and central level for quick action.”* CBH MoH	

##### Challenges to using the DHIS and other reporting tools during crisis

Concerning the reporting circuit, both key informants said that the data managers did not correctly use the DHIS 2. Therefore, the data produced by the system was not useful. Also, the challenges of using paper-based reports in times of crisis were complex because over 40% of facilities are geographically inaccessible even under normal circumstances. During the outbreak, it became even more challenging to have reports because of the insecurities.

**Table:** 

*“For the IDSR we have to detect on time, analyse and share all those things are written in the guideline but to implement we are behind. The reality is that about 40 percent of health facilities are inaccessible so as a government worker during crisis is a problem. If you go alone is a problem of security of you, if you go with the army then the opponent can strike you because they think you are against them.”* EPDRF MoH	

##### Infrastructural, financial, and staffing challenges

Both key informants said that the health staff were a target and that many health workers left, and that most workers on site were natives. This caused a shortage of workers to respond to the outbreak. In addition, partners and the government provided many materials to respond to the outbreak, but how these would be transported to-site was not addressed. Finally, the district and regional level did not have the financial resources to support their decisions which delayed the early alert of the outbreak and contributed to the outbreak's spread.

**Table:** 

*“In terms of resources there were no Pharmacist and staff to take care of patients and staff had to be redeployed to go and support bigger health facilities like the district hospitals. This was because many staff left and only the autochthons staff remained “* CBH MoH “*When bakassi declared the cholera outbreak in 2018, the government and partners donated so much materials but there was no way to transport it to the site. Since they wait for a problem before they donate material therefore it becomes difficult to respond. If the army was not there, we won’t be able to transport the thing.* EPDRF MoH	

## Discussion

By exploring the usefulness of IDSR data in managing the cholera epidemic in a conflict setting, this study is one of the first in Cameroon to approach the problem from a data perspective. The study reveals the need to integrate a complementary surveillance system during the ongoing sociopolitical crisis to enhance data timeliness and completeness. An example of such a system would be the Early Warning Alert and Response Network (EWARN). It was proposed by WHO in 1999 to serve as a parallel surveillance system in public health emergencies and has since been successfully used in conflict-affected settings of Iraq (2013), Lebanon (2006), Pakistan (2005, 2009, 2010), Somalia (2010), Sudan (1999, 2004), and Syria (2012–2013) [17]**.**

Other studies also reported an association between ongoing conflicts and increasing number of cases during outbreaks, even though the transmission dynamics of the outbreaks under investigation were not necessarily the same as that of cholera. During a protracted armed conflict that started in March 2015 in Yemen, Dureab et al*.* in 2019 found an association between increasing cases of diphtheria and the conflict. Correlations have also been reported between the intensity of armed conflicts in Yemen, Syria and Libya and the spread of COVID-19 [18]. Tsung-Shu et al*.* in 2018 found that poor timeliness was responsible for the IDSR system’s inoperativeness. This concurs with the findings from this study in which timeliness was 60% less than the minimum percentage recommended by the national guideline. Metuge et al*.* in 2021, equally highlighted the inability of the IDSR system to provide timely data in the Southwest region of Cameroon, although the scope of the study was limited to community-based surveillance [2]. Other studies in Cameroon have also highlighted timeliness as a major bottleneck to early alert of cholera outbreaks [4, 13], although these were not in the context of armed conflicts.

Our findings suggest that the submission of cholera data during the 2018 outbreak had both external and internal hindrances. For instance, the IDSR guidelines recommend that health facilities report data in paper format. Several health facilities are not geographically accessible, such as Idabato in the Bakassi health district from where the outbreak started. Consequently, it is challenging to submit outbreak data within 24 h as recommended by the national guidelines and poor communication network in facilities further affects data timeliness. External factors such as the sociopolitical crisis led to an atmosphere of insecurity which caused staff to flee the zone, drastically reducing the workforce. Metuge et al., in 2021 reported that data timeliness and completeness during the outbreak was hindered by increased insecurity, shortage of health care staff, and unadapted surveillance systems for conflict situations [2]. However, in the current study, the Kumba Health district and the Buea health districts had average timeliness and completeness of 17.2%; 96.2% and 78.8%; which were comparatively higher than other districts during high conflict intensity. This improvement in reporting could be due to the reallocation of resources to public health emergencies that occurred during the conflict to maintain performance. Both districts are under the Performance-Based Financing (PBF) scheme and thus benefit from some financial autonomy [19] which explains their ability to reallocate and mobilize resources for such emergencies. Also both districts are urban districts and located close to the Regional Delegation for Public Health (RDPH) where reports are submitted. Several studies which examined surveillance systems in times of conflict attributed improved data completeness or timeliness to the presence of complementary systems, such as EWARN [1, 16, 20]. However, in the 2018 cholera outbreak in the Southwest region of Cameroon, this system was not in place and therefore un-influential.

Gaps in data transmission have implications on the district, regional and central levels of the healthcare pyramid. Instructions must be given by the central level before any response [21], primarily because the district lacks financial autonomy. Gaps in data transmission further delay the feedback from the regional and central levels, which will delay timely outbreak response by the district level, increase case fatality and reduce the population’s trust in the health system. At the regional level, data transmission gaps impair the development of a comprehensive risk assessment which can negatively impact the success of the outbreak response. The central level organises rapid response teams, and supports outbreak response activities including communication to the media. Consequently, data transmission gaps could have serious consequences such as general misinformation about the outbreak which could adversely affect outbreak control.

The Sendai Framework for Disaster Risk Reduction highlighted the need for data to be disaggregated and accessible to provide comprehensive knowledge of risks [22, 23]. Arguably, the lack of disaggregated data from the DHIS 2 was an indirect contributor to the IDSR data not being useful for early alert purposes as observed in the current study. The disaggregated data would have provided decision-makers with information on which specific population was at risk of being infected by cholera, and this would have informed need assessment, resource allocation, drafting of a contingency plan, and targeted interventions in case the disease spread to other communities. The current study suggests that disaggregated data could help the IDSR system to be more proactive than reactive in decision-making. Furthermore, although poor data timeliness and completeness in the IDSR system have been consistently reported in previous studies in Cameroon [2, 4, 13, 24], the results from this study suggest that even if reported in time, the data would not be useful if generally incomplete. The data posed more problems for decision-makers because of the time invested in making sense out of the incomplete data during high conflict periods.

Based on the Health Information System Evaluation (HISE) theories of TAM and D&M, the efficiency of the IDSR system at the time of the outbreak failed to deliver because the data generated from the system was not usable and the numerous tools used for reporting demotivated the staff. In addition, the reporting system hindered timely submission of reports because at the time of the cholera outbreak, three parallel reporting systems were used which reduced reporting efficiency (the paper format, the Excel sheet, and the DHIS 2 software). This reduced the reliability of the data especially because the DHIS 2 lacked an internal data validation system to flag incoherences. The acceptance of the DHIS 2 tool by data managers at the time of the outbreak was minimal because they preferred Excel sheets, and the staff were overworked as supported by Asah & Nielsen in 2016. This general situation was exacerbated by the high levels of insecurity, targeting of health personnel, and the geographical inaccessibility of health facilities, which was in line with the findings of Metuge et al*.* in 2021.

It is unclear how the routine IDSR data contributed to the early-warning decision-making process during the cholera outbreak but the system appears to be one that constantly generates data that cannot be used. The system also had a practical inclination to utilize vertical disease surveillance methods rather than the integrated disease surveillance strategy recommended in the national guideline. There was a clear gap between the theoretical standard IDSR guideline and its implementation. The resources invested in the DHIS 2 tool led to the generation of erroneous data, suggesting the crucial need for inter-agency data validations at all levels of the health pyramid before it is shared for actionable decision making. Similar challenges notably, technical, financial and infrastructural which hindered timely reporting of IDSR data were highlighted in the mixed methods study by Tsung-Shu et al. in Malawi in 2018.

This study supports the suggestion of Collins et al*.* in 2020 that numerical data should be accompanied by a narrative piece and that by visualizing the data and reading the narrative, more is covered [24]. Indeed, aspects of context and availability of resources, such as internet connection, information technology literacy and human resource issues, can hinder such a recommendation. However, the combination could provide more specific data with decision-makers better understanding the data collector communication. Ongoing review of the utility of different forms of data communication would help avoid the data generation process being merely mechanical rather than a thoughtful, purposeful and an intentional process suitable for early warning and early action.

This study had some limitations. To determine the association between the timeliness in reporting routine IDSR cholera data and the sociopolitical crisis, the number of deaths per year was used as a proxy to determine conflict intensity. Conflict intensity was categorised as high, medium, and low. Even though this is based on the World Bank classification of conflict intensity, this categorization does not clearly distinguish one category from the next. Therefore, we considered deaths between 150 and 250 as higher intensity and between 20 and 149 as medium intensity. Also, the limitations of using secondary data as an indication of numeric trends was compensated for in the evaluation through key informant interviews that elaborate on the findings from the secondary data.

## Conclusion

The DHIS2 generated disease surveillance data could not be utilized for the early warning of the 2018 cholera outbreak because the data was erroneous. Also, the peripheral level in the health care system collects and enters cholera data into the DHIS2 and does not utilize the data for decision making during public health emergencies. The data produced by the system could be used to monitor disease patterns but could not be used for in-depth analysis and infectious diseases risk data-driven decision-making because of the centralised system. The response to the 2018 cholera outbreak had little use for the data collected and the system seemed to be operational just for reporting purposes. Furthermore, a lack of multiagency data validation and low community participation in cholera risk management were a direct result of the centralized health system which does not provide sufficient collaboration with both local and external partners. Utilizing data at the district level serves as a first-line data quality check which will save time and resources currently directed toward correcting data errors in the DHIS2 platform. This study provides insights on strategies to reduce delays in the utilization of disease surveillance data for informed decision making in conflict settings, notably reinforcing infectious disease risk communication with affected populations through community leaders.

## Data Availability

The datasets used and/or analysed during the current study available from the corresponding author on reasonable request.

## Abbreviations

CBH: Chief Bureau Health
D&M: Delone and MClean Model
DHIMS: District Health Information Management System
DHIS2: District Health Information System 2
DRR: Disaster Risk Reduction
DF/HCC: Dana-Farber/Harvard Cancer Center
EPDRFP: Epidemic prone disease regional focal person
EWARN: Early Warning Alert and Response Network
HIMS: Health Information Management System
HIS: Health Information System
HISE: Health Information System Evaluation
KHD: Kumba District Hospital
MoPH: Ministry of Public Health
NGO: Non-Governmental Organization
NHIC: National Health Information System
RDPH: Regional Delegation for Public Health
TAM: Technology Acceptance Model
UNISDR: United Nation Office for Disaster Risk Reduction
WHO: World Health Organization
